# Microdosimetric Investigation and a Novel Model of Radiosensitization in the Presence of Metallic Nanoparticles

**DOI:** 10.3390/pharmaceutics13122191

**Published:** 2021-12-18

**Authors:** Huagang Yan, David J. Carlson, Ramin Abolfath, Wu Liu

**Affiliations:** 1 School of Biomedical Engineering, Capital Medical University, Beijing 100069, China; yanhg@ccmu.edu.cn; 2 Beijing Key Laboratory of Fundamental Research on Biomechanics in Clinical Application, Capital Medical University, Beijing 100069, China; 3 Department of Radiation Oncology, University of Pennsylvania, Philadelphia, PA 19104, USA; david.carlson@pennmedicine.upenn.edu; 4 Department of Radiation Physics and Oncology, University of Texas MD Anderson Cancer Center, Houston, TX 75031, USA; ramin1.abolfath@gmail.com; 5 Department of Radiation Oncology, New Jersey Urology, West Orange, NJ 07052, USA; 6 Department of Radiation Oncology, Stanford University School of Medicine, Stanford, CA 94305, USA

**Keywords:** nanoparticle, radiosensitization, microdosimetry, theory of dual radiation action, Auger electron, AGuIX, Monte Carlo simulation, proximity function, relative biological effectiveness

## Abstract

Auger cascades generated in high atomic number nanoparticles (NPs) following ionization were considered a potential mechanism for NP radiosensitization. In this work, we investigated the microdosimetric consequences of the Auger cascades using the theory of dual radiation action (TDRA), and we propose the novel Bomb model as a general framework for describing NP-related radiosensitization. When triggered by an ionization event, the Bomb model considers the NPs that are close to a radiation sensitive cellular target, generates dense secondary electrons and kills the cells according to a probability distribution, acting like a “bomb.” TDRA plus a distance model were used as the theoretical basis for calculating the change in *α* of the linear-quadratic survival model and the relative biological effectiveness (RBE). We calculated these quantities for SQ20B and Hela human cancer cells under 250 kVp X-ray irradiation with the presence of gadolinium-based NPs (AGuIX^TM^), and 220 kVp X-ray irradiation with the presence of 50 nm gold NPs (AuNPs), respectively, and compared with existing experimental data. Geant4-based Monte Carlo (MC) simulations were used to (1) generate the electron spectrum and the phase space data of photons entering the NPs and (2) calculate the proximity functions and other related parameters for the TDRA and the Bomb model. The Auger cascade electrons had a greater proximity function than photoelectric and Compton electrons in water by up to 30%, but the resulting increases in *α* were smaller than those derived from experimental data. The calculated RBEs cannot explain the experimental findings. The relative increase in *α* predicted by TDRA was lower than the experimental result by a factor of at least 45 for SQ20B cells with AGuIX under 250 kVp X-ray irradiation, and at least four for Hela cells with AuNPs under 220 kVp X-ray irradiation. The application of the Bomb model to Hela cells with AuNPs under 220 kVp X-ray irradiation indicated that a single ionization event for NPs caused by higher energy photons has a higher probability of killing a cell. NPs that are closer to the cell nucleus are more effective for radiosensitization. Microdosimetric calculations of the RBE for cell death of the Auger electron cascade cannot explain the experimentally observed radiosensitization by AGuIX or AuNP, while the proposed Bomb model is a potential candidate for describing NP-related radiosensitization at low NP concentrations.

## 1. Introduction

Radiosentization by metallic nanoparticles (NPs) has been extensively investigated [1,2,3,4,5,6]. High atomic number (Z) and non-toxic NPs, such as gold (Au, Z = 79) and gadolinium (Gd, Z = 64), are excellent radiosensitizers for kV radiation, as photoelectric absorption cross-sections rise rapidly with decreasing photon energy and increasing Z. The attenuation by high-Z material can be up to 100-fold higher than that of soft tissue. Photoelectrons and Auger cascade electrons are emitted when X-rays interact with high-Z NPs. The Auger electrons have short ranges and can locally damage tumor cells, resulting in a larger biological effect than radiation alone [3]. Even the MV beams normally used in RT, due to the scattering process, contain some percentages of photons and electrons at keV energy as the beams travel deeper into the body [6]. However, it has been known that the averaged macroscopic physical dose increase cannot explain the cell-killing effects [3].

The Auger electron cascade has been considered to have similar behavior to high linear energy transfer (LET) radiation (2–25 keV/μm), inducing high cytotoxicity if the electron source is located in close proximity to the nuclear DNA [1,7,8,9]. Most Auger electrons deposit their energy within 500 nm [7]. In an Auger electron cascade, usually more than one Auger electron is emitted together with the relatively high energy photoelectron. Given their range and temporal and spatial correlation, it is expected that the distribution of distances between energy deposition locations of the electrons will play an important role in the pairwise combination of double-strand breaks (DSBs) caused by these electrons to produce a lethal event (some types of chromosome aberrations) because the spatial proximity of energy transfers governs the combination probability [8].

To explain the dose–response relation for mammalian cells exposed to ionizing radiation, Kellerer et al. developed the theory of dual radiation action (TDRA) [9]. The notion of dual radiation action essentially separates the observable biological effects, represented by lesions, from sublesions (any altered target entities in a cell), which relate directly to energy transfer. It applies to a variety of mechanisms [10,11,12], including the production of a DNA DSB as the result of two strand breaks located on opposite strands [13,14,15]. However, because the formation of DNA DSBs requires damage to DNA strands at a separation no larger than ~3.2 nm [16]—a distance so small that the interaction of energy transfers from separate charged particles can be generally neglected except at doses much higher than those relevant to radiotherapy—DSBs were originally taken as the sublesions in linear-quadratic (LQ) cell survival model, and exchange-type chromosome aberrations were taken as the lethal lesions [9]. With an appropriate distance model, which describes the probability of two sublesions combining into one lesion, the generalized formulation of TDRA [17] is expected to be suitable for analyzing the proximity effects of the energy deposits caused by the multiple electrons from a single photoelectric event.

The TDRA we used is among several biophysical models that describe radiation-induced cell inactivation, e.g., the local effect model (LEM) [18,19], the microdosimetric-kinetic model (MKM) [20], the repair-misrepair (RMR) model [21], the lethal-potentially lethal (LPL) model [22], the repair-misrepair-fixation (RMF) model [23] and the saturated repair model [24]. An LEM has been used to calculate the cell survival fractions in the presence of NPs [2,25,26]. Mathematically, LEM uses the fact that the quadratic terms in the LQ dose response make the high local dose peaks much more important to causing damage. Thus, the inhomogeneity of a dose at the microscopic scale causes a greater effect, which may provide a better description of experimental data. However, the dose inhomogeneity near NPs does not follow an inverse square law or similar law for individual Auger cascades, and the high local dose around NPs is rather a statistical result averaged over many events, unlike the situation of high-LET particle transport in water. Furthermore, LEM cannot properly describe the spatial-temporal correlations among the secondary electrons produced in one ionizing event with NPs. Other mechanistic models of the processing of radiation-induced DNA damage also invoke microdosimetry concepts, such as the MKM and the RMF model [27]. All mechanistic models only produce approximations of the true underlying biological mechanism. We adopted TDRA as a straightforward description of the microdosimetric characteristics of the Auger cascades due to NPs.

In this study, we investigated the biological consequences of the dual radiation actions in the nucleus in the framework of microdosimetry. In particular, we calculated the changes in *α* of the LQ survival model and the relative biological effectiveness (RBE) for cell death based on the proximity distribution of energy deposits due to Auger electrons from NPs. In addition, given the various mechanisms of cell killing enhancement other than the damage due to the Auger cascades [6,21,22,23], we propose a novel phenomenological model to serve as a potential general framework to describe the NP-related radiosensitization.

## 2. Materials and Methods

### 2.1. Generalized Formulation of the Theory of Dual Radiation Action (TDRA)

Here we briefly review the generalized formulation of TDRA [17]. In TDRA, cellular lesions are assumed to be formed as a result of combinations of pairs of sublesions (e.g., chromosome break) in the sensitive sites of the cell. In addition, the yield of sublesions formed within spherical sites is assumed to be proportional to the energy deposited to the site. In the generalized formulation, a distance model was introduced, in which the combination probability, denoted *g*(*x*), depends on *x*, the distance between the pair of the sublesions. In addition, the theory involves two key functions. One is *s*(*x*), the product of the sensitive matrix volume (collectively occupied by the sensitive sites in which sublesions are formed) and the probability density of distances between two points randomly chosen in the matrix, which describes the geometry of the sensitive material in the cell or nucleus. The other is *t_D_*(*x*), the proximity function (or called differential proximity function in some literature), which describes the geometry of the pattern of energy deposition. Specifically, *t_D_*(*x*)d*x* is defined as the expected sum of energy deposited to a shell of radius *x* and thickness d*x* centered at a transfer point.

Given a sublesion and the spherical shell of thickness d*x* and radius *x* centered at it, *s*(*x*)/4π*x*^2^ is the expected fraction of the shell that belongs to the sensitive matrix. The expected number of sublesions in the spherical shell is equal to *ct_D_*(*x*) *s*(*x*)/4π*x*^2^, where *c* is a constant that relates the energy transfer to the yield of sublesions. *t_D_*(*x*) can be separated into a term *t*(*x*) that is independent of *D*, and a second term that is proportional to the absorbed dose. The dose independent term *t*(*x*) stands for the intra-track contribution from the same primary particle, which is affected by radiation quality. The second term represents energy transfers by other, uncorrelated charged particles, i.e.,

(1) $$t_{D}\left(x\right)=t\left(x\right)+4\pi x^{2}\rho D$$

where *ρ* is the density of the irradiated medium. According to its definition, *t*(*x*) can be calculated using the following equation:

(2) $$t\left(x\right)dx=\underset{i}{\sum }\underset{j}{\sum }\varepsilon_{i}\varepsilon_{j}/\underset{i}{\sum }\varepsilon_{i}$$

where *ε_i_* is energy deposited in a single energy transfer, *i* runs over all energy transfer points of a track and *j* runs over all transfer points of the track with a distance in the range [*x*, *x* + d*x*] from the transfer point *i*. A similar method has developed for the analysis of track structures of electrons and protons at the microscopic level through construction of an interface between Geant4-DNA MC and molecular dynamics of DNA and its environment [28].

The yield of lesions can be calculated as follows:

(3) $$N\left(D\right)=\frac{c^{2}\rho V}{2}D\left(\int_{0}^{\infty }\frac{g\left(x\right)t\left(x\right)s\left(x\right)}{4\pi x^{2}}dx+\rho D\int_{0}^{\infty }g\left(x\right)s\left(x\right)dx\right)$$

where *ρ* and *V* are the density and volume of the sensitive matrix (the nucleus in this study), respectively. The equation can also be written as

(4) $$N\left(D\right)=k\left(\xi D+D^{2}\right)$$

where

(5) $$k=\frac{c^{2}\rho^{2}V}{2}\int_{0}^{\infty }g\left(x\right)s\left(x\right)dx$$

and

(6) $$\xi =\int_{0}^{\infty }\frac{g\left(x\right)t\left(x\right)s\left(x\right)}{4\pi \rho x^{2}}dx/\int_{0}^{\infty }g\left(x\right)s\left(x\right)dx$$

*ξ* reflects the radiation quality. The interaction probability *g*(*x*) can be modelled as [17]

(7) $$g\left(x\right)=Ce^{-x/a}$$

where *a* and *C* are constants that depend on the cell line and phase, and *a* describes the decay of interaction probability over distance. *C* is the probability of the combination of two sublesions when they are at the same location. As *g*(*x*) always comes together with the cell-line dependent parameter *c*, which is determined from experimental data, the exact value of *C* has little impact on the results. In our calculations, we assumed *C* = 1, and *a* would be determined from fitting data of cell experiments.

### 2.2. The Application of TDRA to NP Radiosensitization

In the presence of NPs, the yield of lesions can be divided into three parts, those combined from sublesions caused by secondary electrons due to NPs (*N*_1_(*D*)), exclusively due to water (*N*_2_(*D*)) and from sublesions by secondary electrons due to NPs and water (*N*_3_(*D*)). For clinically relevant concentrations we considered, the combination of sublesions caused by secondary electrons from different NPs could be neglected. As the number of photons entering an NP is proportional to the dose to the surrounding water without the NPs, let *λ* be the proportionality coefficient, then the number of photons entering an NP equals *λD*. Consequently, the probability of an ionization event in an NP is *μλD*, where *μ* is the probability of an ionization event in the NP when only one photon enters the NP. If there are *n* NPs in a cell, then the expected number of photon ionization events in the cell due to the NPs is *nμλD*. Suppose the energy deposited in the nucleus by all secondary electrons from an ionization event in an NP is *E* (which is essentially the product of nucleus mass and the specific energy (*z*) to the nucleus, i.e., *E* = *m*_nucleus_ · *z*), then

(8) $$N_{1}\left(D\right)=\frac{c^{2}n\mu \lambda }{2}D\int_{0}^{\infty }\int_{0}^{\infty }\frac{Ep\left(E\right)g\left(x\right)t_{\mathrm{NP}}\left(x,E\right)s\left(x\right)}{4\pi x^{2}}dxdE$$

(9) $$N_{2}\left(D\right)=\frac{c^{2}\rho V}{2}D\left(\int_{0}^{\infty }\frac{g\left(x\right)t\left(x\right)s\left(x\right)}{4\pi x^{2}}dx+\rho D\int_{0}^{\infty }g\left(x\right)s\left(x\right)dx\right)$$

(10) $$N_{3}\left(D\right)=c^{2}n\mu \lambda \rho D^{2}\int_{0}^{\infty }Ep\left(E\right)dE\int_{0}^{\infty }g\left(x\right)s\left(x\right)dx$$

where *t*_NP_(*x*, *E*) is calculated from all energy transfer points inside the nucleus, no matter whether the NP is inside or outside—the total energy deposit in the nucleus is *E* and *t*(*x*) is the proximity function of the electrons from water molecules. *p*(*E*)*dE* is the probability of energy deposit in the nucleus within the range [*E*, *E* + *dE*] due to a photon ionizing event in an NP, which depends on the track structure of the secondary electrons and the distribution of NPs in the cell and the nucleus size.

In this study, we assumed that targets are randomly distributed in the nucleus. Therefore, *s*(*x*)/4π*x*^2^ is regarded constant in the nucleus and equals the ratio of effective DNA volume and the nucleus volume, which varies among cell lines and is denoted as *η* in the following derivations. Using Equation (7) and assuming *C* is 1, we have

(11) $$N_{1}\left(D\right)=\frac{\eta c^{2}n\mu \lambda }{2}D\int_{0}^{\infty }\int_{0}^{\infty }Ep\left(E\right)e^{-x/a}t_{\mathrm{NP}}\left(x,E\right)dxdE$$

(12) $$N_{2}\left(D\right)=\frac{\eta c^{2}\rho V}{2}D\left(\int_{0}^{\infty }t\left(x\right)e^{-x/a}dx+8\pi a^{3}\rho D\right)$$

(13) $$N_{3}\left(D\right)=8\pi c^{2}a^{3}n\mu \lambda \rho \eta \bar{E}D^{2}$$

where

$$\bar{E}=\int_{0}^{\infty }Ep\left(E\right)dE$$

is the average total energy deposit to the nucleus per ionization in the NPs.

If *N*_2_(*D*) takes the form of Equation (4), i.e.,

(14) $$N_{2}\left(D\right)=k\left(\xi D+D^{2}\right)$$

Then

(15) $$k=4\pi \eta c^{2}\rho^{2}Va^{3}$$

(16) $$\xi =\frac{\int_{0}^{\infty }t\left(x\right)e^{-x/a}dx}{8\pi a^{3}\rho }$$

On the other hand, *ξ* can be calculated from experimentally obtained *α*/*β* ratio for a particular cell type and a set of irradiation conditions. Therefore, we could use this experimental *ξ* to determine the value of *a* based on Equation (16) and *t*(*x*), which for multienergetic electrons can be calculated from proximity function of monoenergetic electrons with energy weighted spectrum [29]. With the presence of NPs, the expected total yield of lesions becomes

(17) $$N\left(D\right)=N_{1}\left(D\right)+N_{2}\left(D\right)+N_{3}\left(D\right)=k\left(\xi^{\prime }D+D^{2}+\zeta D^{2}\right)$$

where

(18) $$\xi^{\prime }=\xi +\Delta \xi =\xi +\frac{n\mu \lambda \int_{0}^{\infty }\int_{0}^{\infty }Ep\left(E\right)t_{\mathrm{NP}}\left(x,E\right)e^{-x/a}dxdE}{8\pi a^{3}\rho^{2}V}$$

$$\zeta =\frac{n\mu \lambda \bar{E}}{\rho V}$$

The RBE of the NPs, relative to photon beam irradiation alone, is defined as $R=\frac{D}{D_{\mathrm{NP}}}$, where *D* and *D*_NP_ denote the doses absorbed by the water without and with NPs that cause equal biological effects. It follows that

(19) $$R=\frac{1}{2D_{\mathrm{NP}}}\left[\sqrt{\xi^{2}+4D_{\mathrm{NP}}\left(\xi^{\prime }+D_{\mathrm{NP}}+\zeta D_{\mathrm{NP}}\right)}-\xi \right]$$

### 2.3. A Novel Phenomenological Model—Bomb Model

To describe the radiosensitizing effect of NPs with X-ray irradiation, we propose a phenomenological model that can incorporate a variety of unknown mechanisms of NP radiosensitization. The model is named Bomb model for its simple characteristics. Essentially, the model regards an NP as a bomb that can be triggered by a photoelectric interaction/Compton scattering and kill the cell at a certain probability. The probability depends on the type of NP, the cell line and phase, the location of the NP inside the cell and the energy of the irradiating X-ray. For a particular NP type and cell line and phase, suppose that the number of NPs outside the nucleus is *N*_1_ and the number inside is *N*_2_, and the corresponding probabilities of cell killing are *p*_1_ and *p*_2_, respectively; and ignore the additional physical dose due to the NPs. The expected number of lethal events in a cell added by irradiated NPs would be

$$N_{\mathrm{NP}}\approx N_{\mathrm{ioni}1}\cdot p_{1}+N_{\mathrm{ioni}2}\cdot p_{2}\approx N_{1}\cdot p_{1\mathrm{Gy}}\cdot D\cdot p_{1}+N_{2}\cdot p_{1\mathrm{Gy}}\cdot D\cdot p_{2}=\Delta \alpha \cdot D$$

(20) $$\Delta \alpha =p_{1\mathrm{Gy}}(N_{1}\cdot p_{1}+N_{2}\cdot p_{2})$$

where *N*_ioni1_ and *N*_ioni2_ denote the numbers of ionizations in all NPs in the cytoplasm and in the nucleus, respectively; and $p_{1\mathrm{Gy}}$ is the probability of one ionizing event in one NP when the dose to the surrounding water is 1 Gy, which equals the product of *μ* and *λ*. Here the probability of two or more ionizations in one NP is neglected, because it is generally much less than $p_{1\mathrm{Gy}}$. According to Equation (20), the radiosensitizing effect of NPs can be quantitatively described by an increase in *α* (the linear term) of the LQ model of the survival curve. As the model is intended to describe the local effect of NPs to the cell, it should be noted that it is applicable only when the physical dose added by the NPs is small compared with the X-ray dose to the surrounding water. Otherwise, the enhanced dose should also be considered. This model assumes a higher biological effect of the NP + radiation + ionization-within-NP combination than the physical dose enhancement alone. It is aimed to address the experimentally observed radiation damage enhancement at relatively low NP concentration, which cannot be explained by the physical dose enhancement alone. The relatively low NP concentration (e.g., on the order of 0.1 mM in Gd for AGuIX) is in the clinically feasible range for human applications, similar to that used clinically for diagnostic imaging. Many simulation studies assumed much higher NP concentrations that are not easily achievable in clinic.

Although currently it is impossible to determine *p*_1_ and *p*_2_ theoretically, the model provides testable predictions on the upper limit of change in *α* if the concentration of NP in a cell can be measured, because both *p*_1_ and *p*_2_ are less than 1, and $p_{1\mathrm{Gy}}$ can be determined using a Monte Carlo (MC) simulation. In this study, based on the measured *α* values, we calculated the *p*_1_ for the four sets of irradiation conditions of Hela cells described in Chithrani et al. [30], one set of irradiation condition of SQ20B cells described in Miladi et al. [31] and one set of irradiation condition with two incubation concentrations of A549 cells described by Liu et al. [32].

### 2.4. The Nanoparticles and the Cell Models

In this study, a Gadolinium-based NP, AGuIX^®^ [33,34], (~3 nm diameter) and an AuNP [30] 50 nm in diameter were investigated. There are on average 10 Gadolinium atoms on each AGuIX NP. The atomic mass of an AGuIX is approximately 9 kDa. The size of 50 nm was chosen for AuNP because Chithrani et al. [35] found the maximum uptake of AuNPs by Hela cells occurred at an NP size of 50 nm. For simplicity, in the MC calculations both NPs were modeled as a uniform sphere, with a diameter of 3.0 nm and density of 1.2 g/cm^3^ for AGuIX, and 50 nm and 19.32 g/cm^3^ for the AuNP.

**Figure 1:**
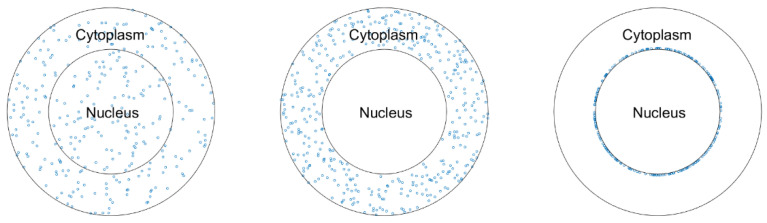
Three scenarios of NP distribution in the cell.

We studied three human cancer cell lines—Hela, SQ20B and A549—with existing experimental data. Simplified models with spherical cell and nucleus volumes were used for all the cells. The typical radii of a SQ20B cell and its nucleus are approximately 10.6 μm and 8.1 μm, respectively [36], and the corresponding values for a Hela cell are 8.6 μm [37] and 5.5 μm [38], respectively. The typical volumes of a A549 cell and its nucleus are approximately 1670 μm^3^ and 466 μm^3^, respectively [39]. Therefore, a nucleus radius of 4.8 μm and a cell radius of 7.4 μm were used for the A549 cell. To quantify NP radiosensitization, three scenarios of NP distribution in the cell were considered (Figure 1) (a) uniformly distributed throughout the entire cell, (b) uniformly distributed in the cytoplasm only, and (c) uniformly distributed around the nuclear membrane. The third scenario was simulated because some researchers found that NPs were located around the nuclei [30,40]. As we only focused on the local radiation effects of NPs, the effect of NPs outside the cell was not considered.

### 2.5. MC Simulation of Irradiation on NP and Secondary Electrons Transport

Geant4 [41] (version 10.7.2) and its extension Geant4-DNA [42,43,44,45] were used for our MC simulations. The MC simulations for this study can be divided into four steps. In Step 1, the irradiation of a cell centered at a water cylinder with a photon beam was simulated. The purpose is (a) to determine the average number of photons entering an NP when the dose to the surrounding water is 1 Gy, i.e., the parameter of *λ* described in Section 2.2; (b) to collect the phase-space data of photons entering a sphere representing the NP, which will be used in later simulations; and (c) to determine the spectrum of secondary electrons generated in the NP-representing sphere. To compare with existing experiment results, we used two X-ray sources for the microdosimetric investigation of Auger cascade, 220 kVp for Hela cell with AuNPs, and 250 kVp for SQ20B cell with AGuIX; we also used three additional X-ray sources for the calculation of the Bomb model parameters: 105 kVp, ^137^Cs (660 kev) and 6 MV. All photon beams were directed uniformly from the top of the water cylinders, and the height and diameter of which were 1.0 mm, 2.0 mm, 2.0 mm, 20 mm, and 30 mm, respectively for the five energies (105 kVp, 220 kVp, 250 kVp, ^137^Cs (660 keV) and 6 MV), to allow electron build-up at the depths of the cells.

In Step 2, the spectrum of the secondary electrons was used to simulate transport of secondary electrons of orthovoltage radiotherapy in water and the proximity functions were computed using a method described by Incerti et al. [46]. The study of the Bomb model does not involve this step.

In Step 3, the phase-space data collected in Step 1 were used as virtual sources to irradiate the NPs. As the sphere on which the phase-space data were collected in Step 1 had a radius of 50 μm to obtain sufficient count of photons, they were used in this step after scaling down the position coordinates such that the photons were all directed toward the NPs. The purpose of this step is to obtain the probability of an ionization event per photon entering the NPs, i.e., the parameter of *μ* described in Section 2.2.

**Figure 2:**
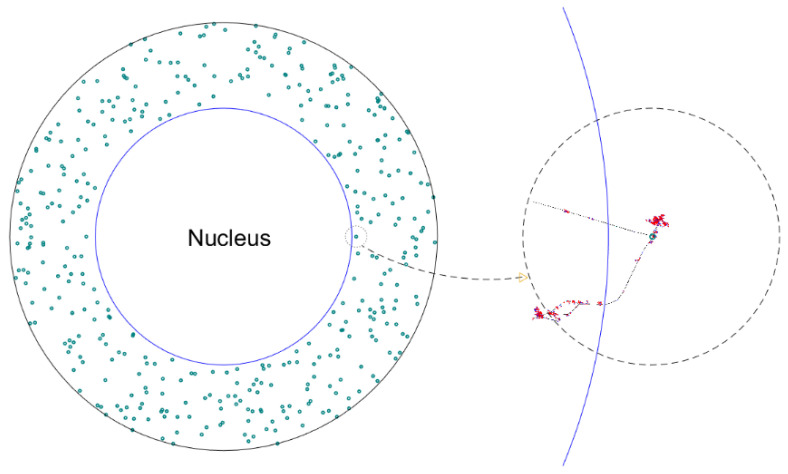
Scoring of the energy deposits in the nucleus. The right dashed circle is a magnified view of the small dotted circle on the left with an NP at the center. It represents an ionized NP in the cytoplasm. To score the energy deposits by the electrons from the NPs, each ionized NP is fixed to the origin in the simulation, and the volume of the nucleus, i.e., the volume that has energy deposits scored, is translated accordingly such that the position of the NP relative to the nucleus is kept the same. In the plot, the blue circle represents the boundary of the nucleus.

In Step 4, which was required only for the microdosimetric study, the phase-space data were used again as in Step 3 to irradiate the NPs. The track structure of the secondary electrons from the NPs was analyzed, and the proximity functions of all secondary electrons from an ionization of NPs were calculated using a method similar to that used in Step 2. To simulate the irradiation of the three scenarios of NP distribution mentioned above, the NPs were dynamically and randomly distributed in the whole cell, in the cytoplasm, or on the nuclear membrane, and randomly chosen photons from the phase space data were directed at the NPs accordingly. For convenience, in the simulations, the NPs were fixed to the origin in the coordinate system of Geant4, as illustrated on the right of Figure 2. The energy deposits in the nucleus were collected for proximity function calculation. Due to the small size of the NP, the probability of photoelectric interaction and Compton scattering between a photon and an NP is very small. To improve the efficiency of computation, the interaction cross-sections of photoelectric effect and Compton scattering in the NPs were amplified by a factor of 50,000 for AGuIX and 20 for AuNP, and all photons after one interaction in the NPs were killed to avoid bias of secondary interaction.

Livermore physics was used for the interaction of photons with water and the NP, and Geant4-DNA option 2 was used for the track structure simulation of the secondary electron in water in Step 2 and Step 4. The tracking cutoff 7.4 eV was applied for electron transport in water.

Additional technical details of the simulations are given in Table A1 in Appendix A.

### 2.6. Postprocessing of the Results from the MC Simulations

To calculate the proximity functions, Equation (2) was used to process the energy deposition data from Step 2 and Step 4. Equation (16) was then used to determine parameter *a* of the distance model based on the proximity functions calculated from Step 2 and *ξ* derived from experimental data. However, the results of *α* and *β*, which were obtained by fitting experimental data to a LQ survival curve, vary among studies for SQ20B cells under 250 kVp X-ray irradiation. We took the smaller *ξ* (0.8 Gy) calculated from the *α* and *β* obtained by Miladi et al. [31] as the lower limit of *ξ,* and the larger *ξ* (2.33 Gy) calculated based on Wozny et al. [47] as the upper limit. For Hela cells under irradiation of 220 kVp X-rays, the uncertainty information of *α* and *β* has been provided by Chithrani et al. [30].

After calculating the proximity functions (*t*_NP_(*x*, *E*)) of electrons from individual ionizations in the NPs and the corresponding *E*’s from the output of Step 4, Equation (18) was used to calculate the change in *ξ* based on the number of NPs per cell (*n*), and the results of *λ* and *μ* from Step 1 and Step 3, respectively. The increases in *α* and RBE were calculated afterwards.

As the investigation of Bomb model did not involve proximity function, it used *n*, *λ* and *μ* only.

## 3. Results

### 3.1. Parameters λ and μ

The average number of photons entering an NP per Gray of dose to the surrounding water, *λ*, and the probability of an ionization event per photon entering the NPs, *μ*, were determined based on the simulations of Step 1 and Step 3. Table 1 lists the results of *λ* and *μ*, together with their product $p_{1\mathrm{Gy}}$.

### 3.2. The Spectrum of Energy Deposited in the Nucleus and the Proximity Function

**Figure 3:**
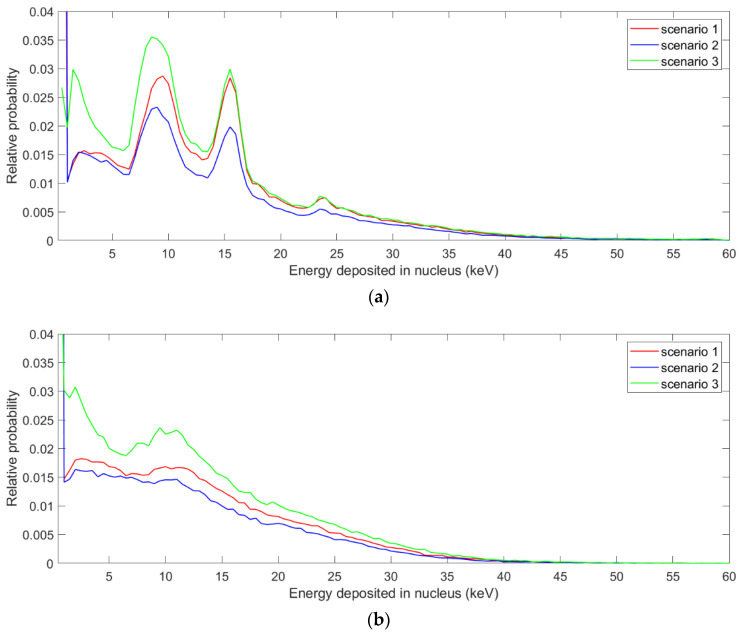
The distribution of energy deposited in the nucleus from one photon ionizing event for AGuIXs (**a**) and AuNPs (**b**), irradiated with 250 kVp and 220 kVp beams, respectively.

**Figure 4:**
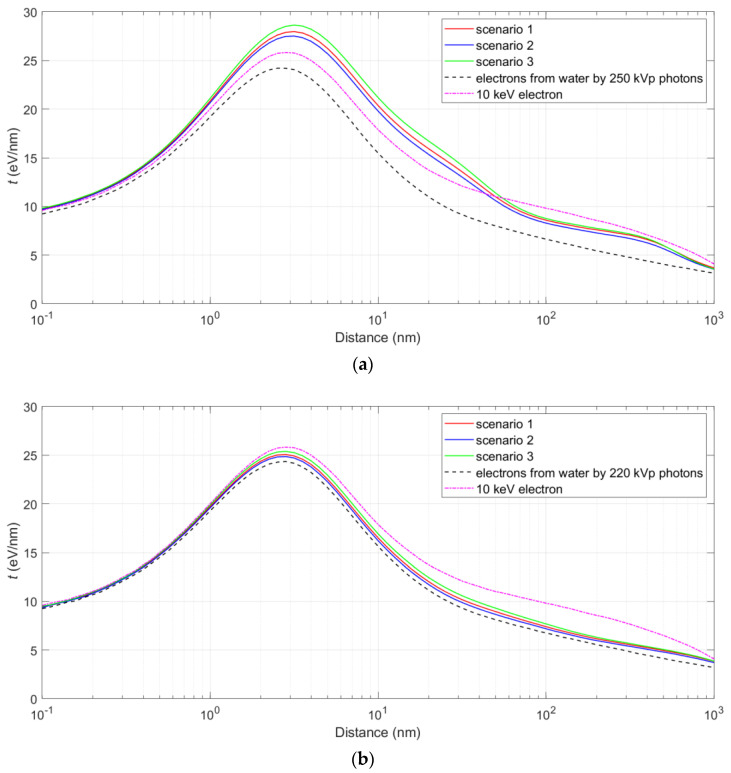
Energy weighted proximity functions of the secondary electrons produced from water at 1 mm depth, and the proximity functions of the electrons in the nucleus due to AGuIXs (**a**) and AuNPs (**b**) for the three scenarios.

Figure 3 shows the distribution of energy deposited in the nucleus from one photon ionizing event for NPs. Note that in scenarios 1 and 2, there is a considerable probability that the energy deposit in the nucleus is zero, because either large number of NPs or all NPs are outside the nucleus. In scenario 3, this probability is smaller, because the NPs are close to the nucleus and the Auger electrons tend to distribute in all directions. From this distribution, the average specific energies to the SQ20B nucleus per ionization in AGuIX with the irradiation of a 250 kVp beam were calculated to be 8.2 × 10^−4^, 6.4 × 10^−4^ and 9.1 × 10^−4^ Gy for the three scenarios, respectively. The corresponding results for AuNP in Hela cells with the irradiation of the 220 kVp beam were 2.0 × 10^−3^, 1.7 × 10^−3^ and 2.6 × 10^−3^ Gy for the three scenarios, respectively. Figure 4 shows the energy weighted proximity functions of the secondary electrons produced from water at 1.0 mm depth and the proximity functions of the electrons in the nucleus due to NPs for the three scenarios. The proximity function of 10 keV electrons is also presented for comparison.

### 3.3. Parameter a of the Distance Model, and the Changes in α and RBE

From the proximity function of secondary electrons calculated from Step 2, the lower and upper values of parameter *a* of the distance model for the SQ20B cells with AGuIX under the irradiation of the 250 kVp beam were determined to be 0.147 and 0.234 μm, respectively, corresponding to the larger *ξ* based on Wozny et al. [47] and the smaller *ξ* based on Miladi et al. [31]. The value of *a* for the Hela cells with 50 nm AuNP under 220 kVp photon irradiation was 0.122 μm. It should be noted here that *a* depends on the cell line and its phase. It is independent of the radiation (and hence NP type) used.

The calculated increases in *α* and results of RBE are shown in Table 2. To calculate Δ*ξ*, the number of AGuIXs in a cell was determined based on the intracellular Gd concentration, which was taken to be 0.10%, corresponding to approximately 10 times the incubation Gd concentration of 0.6 mM, which was measured by Rima et al. [48]. This concentration gives about 6.06 × 10^8^ NPs of AGuIX in a typical SQ20B cell. The number of 50 nm AuNPs per Hela cell was taken to be 6000 according to the measurement by Chithrani et al. [35]. For comparison, the dose enhancement ratio (DER), defined as the ratio of total nucleus dose (dose to the surrounding water + average specific energy to the nucleus by the NPs) to the dose to the surrounding water, was also calculated.

### 3.4. Predictions by the Bomb Model for the Radiosensitization

Since no NPs have been seen to internalize into the cell nucleus [49,50], we assume *N*_2_ = 0 in Equation (20) and are left with one parameter, *p*_1_, the probability of cell killing due to an ionization by NPs outside the nucleus. The results of *p*_1_ together with other parameters of Equation (20), are shown in Table 3 for Hela, SQ20B and A549 cells irradiated with photon beams. Although the *p*_1_ calculated from the experiments might be subject to uncertainties other than those that were considered in the experiment and in our MC simulations—for example, the influence of NPs on the cell membrane—the results do show a trend that NPs ionized by photons of higher energy have higher killing potential. One estimate of *p*_1_ is slightly more than 1. This could be caused by the inaccuracy of the experimental data or spectral data, or by the inconsistency of irradiation settings between the MC simulation and experiment.

## 4. Discussion

### 4.1. Study Limitations and Implications of the Microdosimetric Investigation

In this study, the radiosensitization of a Gd-based NP, known as AGuIX, and AuNP, was analyzed in the framework of microdosimetry using a proximity function and based on the theory of dual radiation action. RBE was calculated for three scenarios of NP distribution in the cell. We found that the biological effect due to the spatial correlations between Auger electrons in a single ionizing event in NP is not significantly more substantial than that calculated from physical dose enhancement. This implies that although the electrons in the Auger cascade following an ionizing event in NPs have been viewed as high-LET particles, their impact is overestimated, because their energies are generally low and the proximity function at 1–50 nm is only 10–20% greater than, or even less than that of an exemplary 10 keV electron (see Figure 4). In contrast, the proximity function for a 100 keV proton is more than four times higher than that of the 10 keV electron over the range 0.1–500 nm. In other words, the density of energy deposition by secondary electrons from NP—including Auger electrons—is not high enough to give an RBE that is consistent with experimental observations. For example, the RBE for scenario 3 at 2 Gy predicted by TDRA and that from the experiment were 1.02 and 1.61 (based on Wozny et al. [47]) for SQ20B cells with AGuIX under 250 kVp X-ray irradiation.

From the comparison of the three scenarios of NP distribution inside the cell, the closer the NPs to the nucleus, the greater the RBE. For instance, while the RBE was 1.25 in scenario 2 for Hela cells with AuNP at 2 Gy, the RBE for scenario 3 was 1.38. The difference in Δ*α* was even more significant. The value for scenario 3 could be 1.57 times the value for scenario 2. This confirms the expected effect of NPs’ distance to the DNA for cell killing and the importance of delivering the NPs into cancer cells for therapy, as even if there are no NPs in the nucleus, the RBE could still be considerable, especially if the NPs are near the nucleus.

We used the average NP number per cell in our calculation. It was observed that cellular uptake of NPs may vary by orders of magnitude among cells [32]. This variation could affect the survival characteristics but may be challenging to measure. We did not consider free radicals and DNA repair. Furthermore, many studies found that the chemical properties of NP may play an important role in their radiosensitizing effect, and there may be other mechanisms of cell killing than direct DNA damage [6,52,53,54].

SQ20B is a radioresistant cell line. Based on TDRA and its low *α*/*β* value of ~1–2 Gy [31,47], the killing effect by photon irradiation must be achieved mainly by inter-track interactions, even at a few Gy, which means that the contribution of cell killing from intra-track interactions brought by Auger electrons could be less important. This may partly explain the low RBE calculated from TDRA. However, a substantial radiosensitizing effect was observed for SQ20B cells incubated by AGuIX [31], which suggests that mechanisms other than the Auger cascade, such as the catalytic effect of the NP surface [53], might also be involved.

Goodhead et al. [55] suggested that the biological effect of low-LET radiation was predominantly due to its track-end clustered ionizations, and Nikjoo et al. [56] suggested that for electron and photon irradiation, clusters of energy depositions are mainly due to low energy track-ends, and nearly 20–30% of the DSBs are complex (additional breaks within the range of 10 bp) for these low energy electrons (100 eV to ~5 keV). The RBE of many endpoints due to the track-ends would therefore be higher than that of far more sparse ionizations. Nonetheless, as the proximity function at short distances reflects clustering of energy transfers [57], the small difference (<25%) in the proximity function values around or less than 1 nm between the secondary electrons with NPs and without NPs implies that the RBE due to the NPs is not large. Therefore, the general conclusion of our microdosimetric investigation could remain unchanged if complex types of DNA lesions produced by multiple track ends are considered. The issue of complex types of DNA lesions has been discussed in our previous work [23].

### 4.2. Implications of the Bomb Model

One direct prediction from the Bomb model is that NPs enhance radiation potency only by increasing the *α* in the LQ survival model. Table 4 lists a few experimental studies of NP radiosensitization that reported data on *α* and *β*. *β* in general changes little with the presence of NPs, which was also observed by Lux et al. [58].

From the comparison of *p*_1_ (probability of cell killing per ionization in NP) between AuNPs and AGuIXs, we can see that the ionization of AGuIXs provides a greater killing potential than that of AuNP at approximately the same photon energy. This is because at the same mass of NPs per cell, the larger NPs tend to absorb more secondary electrons within them, especially for low energy electrons; thus, less are left to damage the cells. Chithrani et al. [35] compared the intracellular uptake of different sized and shaped colloidal AuNPs and found the maximum uptake of AuNPs by Hela cells occurred at an NP size of 50 nm. Studies on AGuIX [40,54], TiO_2_ [49] and AuNPs [50] did not detect NPs in cell nuclei. However, Huo et al. [59] found gold NPs smaller than 6 nm may enter the nucleus.

In Table 3, the two lines for “A549” and “AGuIX” show the results of A549 cells incubated with AGuIX and AGuIX conjugated with pH-low insertion peptide (pHLIP) [32]. The latter can significantly increase the cellular uptake of the NPs (*N*_1_) in accordance with Equation (20), which results in a more significantly increased *α*, indicating enhanced radiosensitivity.

Regardless of the underlying mechanism of radiosensitization, if the cell killing probability *p*_1_ can be estimated using Equation (20) based on the increase in *α*, which is fitted from experimental observations, and the intra-cellular NP concentration can be determined via imaging or other means, the model can be used to guide treatment planning, as the RBE can be calculated directly from the increase in *α* based on the *α* and *β* in the LQ survival model, similarly to the calculation of Equation (19). For example, for Hela cells with 50 nm AuNPs irradiated by 220 kVp beams, the increase in *α* is 0.202 Gy^−1^ when there are on average 6000 AuNPs per cell; the corresponding RBE would be 1.56 at 2 Gy. According to the Bomb model, the RBE at 2 Gy increases to 2.02 if the number of AuNPs per cell doubles. In addition, the comparison of *p*_1_ among different cell lines, NP types or irradiating photon energies can provide us an insight into the nature of NP radiosensitization. For instance, Table 3 shows that *p*_1_ increases monotonously for Hela cell with AuNPs when the energy of irradiating photons increases, which implies that NPs ionized by photons of higher energies have higher killing power, and because for an MV beam, the Compton scattering in NP dominates over the photoelectric effect, it can have a stronger effect than the photoelectric effect, even though the latter can result in the Auger cascade.

More quantitative and comprehensive testing of the Bomb model requires more accurate measurement of the intracellular uptake of NPs, which will be investigated in the future. As *p*_1_ would need to be determined for each individual tumor and for each type of NPs if the model were to be applied in clinical treatment, accumulating knowledge of the approximate range of the *p*_1_ values in various conditions should be achieved with more experimental data.

## 5. Conclusions

The RBE calculated based on the TDRA and proximity function of NP-generated Auger cascade electrons for cell damage cannot explain the experimentally observed radiosensitization by AGuIX under irradiation of 250 kVp X-rays but may partially explain the change in *α* observed in Hela cells with AuNPs under irradiation of 220 kVp X-rays. The radiosensitivity strongly depends on the distance from and spatial distribution of the NPs in relation to the nucleus. The proposed Bomb model may be a potential candidate for a general framework that describes radiosensitization by NPs.

## Figures and Tables

**Table 1:** Results of *λ*, *μ*, and $p_{1\mathrm{Gy}}$.

NP	Photon Beam	*λ* (Photons per Gy per NP)	*μ* (Ionizations per Photon)	$p_{1Gy}$ (Ionizations per Gy per NP)
AGuIX	250 kVp	0.168 ± 0.003	(3.00 ± 0.06) × 10^−7^	(5.04 ± 0.14) × 10^−8^
50 nm AuNP	105 kVp	46.1 ± 0.9	(6.41 ± 0.13) × 10^−4^	(2.96 ± 0.08) × 10^−2^
	220 kVp	47.7 ± 1.0	(4.09 ± 0.08) × 10^−4^	(1.95 ± 0.05) × 10^−2^
	^137^Cs (660 keV)	5.97 ± 0.12	(8.96 ± 0.18) × 10^−6^	(5.35 ± 0.15) × 10^−5^
	6 MV	2.63 ± 0.05	(4.63 ± 0.09) × 10^−6^	(1.22 ± 0.03) × 10^−5^

**Table 2:** Relative theoretical and measured increases in *α*, RBEs and DERs due to NPs for different scenarios of NP distribution in the cells at reported and hypothetical NP concentrations.

NP	Scenarios of NP Distribution	Concentration (# per Cell)	Δ*ξ* (Gy)	Δ*α*_cal_/*α* ^a^	Δ*α*_exp_/*α*	RBE at 2 Gy	DER ^b^
AGuIX (3 nm)	1	6.06 × 10^8^	0.027–0.078	0.034	1.7–11	1.016–1.020	1.025
		6.06 × 10^9^	0.27–0.78	0.34		1.15–1.19	1.25
	2	6.06 × 10^8^	0.020–0.059	0.025		1.012–1.015	1.019
		6.06 × 10^9^	0.20–0.59	0.25		1.12–1.15	1.19
	3	6.06 × 10^8^	0.031–0.089	0.038		1.017–1.022	1.027
		6.06 × 10^9^	0.31–0.89	0.38		1.16–1.21	1.27
50 nm AuNP	1	6000	0.93	0.25	1.35	1.31	1.24
		18,000	2.8	0.76		1.76	1.71
	2	6000	0.75	0.21		1.25	1.20
		18,000	2.3	0.62		1.64	1.59
	3	6000	1.20	0.33		1.38	1.30
		18,000	3.6	0.98		1.93	1.91

Note: ^a^ The relative increases in *α* were calculated from Δ*ξ* and *ξ*; ^b^ Dose enhancement ratio $DER\equiv \frac{D_{\mathrm{NP}}}{D_{W}}=\frac{D_{W}+\Delta D}{D_{W}}$, where Δ*D* is the dose deposited in the nucleus by all ionized NPs in the cell, and *D_W_* is the dose given to the surrounding water.

**Table 3:** Bomb model parameters for the cell irradiation with photon beams.

Cell and NP	Irradiation Photons	# of NPs per Cell	*α* without NPs (Gy^−1^)	*α* with NPs (Gy^−1^)	*p*_1_	Survival Fraction (SF) at 2Gy without NPs	Survival Fraction (SF) at 2Gy with NPs	RBE at 2Gy
SQ20B, AGuIX	250 kVp	6.06 × 10^8^	0.04	0.5	0.015 ^a^	0.76	0.33	2.17
A549, AGuIX		1.66 × 10^7^	0.332 ± 0.045 [51]	0.349 ± 0.054 ^b^	0–5.6 × 10^−2^	0.48	0.46	1.04
		1.32 × 10^9^		0.488 ± 0.063 ^b^	(2.34 ± 0.67) × 10^−3^		0.35	1.37
Hela, AuNP	105 kVp	6000	0.237 ± 0.005	0.528 ± 0.007	(1.64 ± 0.04) × 10^−3^	0.53	0.28	1.69
	220 kVp	6000	0.150 ± 0.004	0.352 ± 0.005	(1.73 ± 0.05) × 10^−3^	0.63	0.42	1.56
	^137^Cs (660 keV)	6000	0.119 ± 0.013	0.259 ± 0.011	0.436 ± 0.055	0.67	0.53	1.39
	6 MV	6000	0.110 ± 0.008	0.191 ± 0.002	1.11 ± 0.12	0.71	0.60	1.35

Note: ^a^ The experimental data from Miladi et al. [31] did not have uncertainty info; therefore, the corresponding uncertainty in *p*_1_ is not given. ^b^ The survival data from Liu et al. [32] were not complete, and the change in *α* was calculated by assuming no change in *β* (*β* = 0.018 Gy^−2^ according to Wera et al. [51]).

**Table 4:** Changes in *α* and *β* reported by some literature on NP radiosensitization.

References	NP Type and Concentration	Radiation (Photons)	Cell Type	Change in *α* (Gy^−1^)	Change in *β* (Gy^−2^)
Chithrani et al. [30]	50 nm Gold NP, 6000 NPs per cell,	105 kVp	HeLa	0.237 to 0.528	0.041 to 0.054
		220 kVp		0.150 to 0.352	0.041 to 0.041
		^137^Cs (660 keV)		0.119 to 0.259	0.040 to 0.030
		6 MVp		0.110 to 0.191	0.029 to 0.031
Jain et al. [60]	1.9 nm Gold NP, 12 μM	160 kVp	MDA-MB-231	0.019 to 0.091	0.052 to 0.093
		6 MV		0.002 to 0.104	0.079 to 0.098
		15 MV		0.083 to 0.061	0.059 to 0.121
Butterworth et al. [61]	1.9 nm Gold NP, 10 μg/mL^−1^	160 kVp	AGO-1552B	0.25 to 0.30	0.04 to 0.05
			Astro	0.37 to 0.40	0.08 to 0.09
			DU-145	0.03 to 0.05	0.04 to 0.04
			L132	0.12 to 0.11	0.03 to 0.03
			MCF-7	0.46 to 0.28	0.02 to 0.07
			MDA-231-MB	0.09 to 0.15	0.03 to 0.03
			PC-3	0.12 to 0.29	0.06 to 0.03
			T98G	0.04 to 0.14	0.03 to 0.02
	1.9 nm Gold NP, 100 μg/ml		AGO-1552B	0.25 to 0.68	0.04 to <0.04
			Astro	0.37 to 0.23	0.08 to 0.16
			DU-145	0.03 to 0.04	0.04 to 0.04
			L132	0.12 to 0.05	0.03 to 0.04
			MCF-7	0.46 to 0.24	0.02 to 0.08
			MDA-231-MB	0.09 to 0.27	0.03 to 0.02
			PC-3	0.12 to 0.21	0.06 to 0.03
			T98G	0.04 to 0.06	0.03 to 0.02
Stefancikova et al. [40]	AGuIX, 0.5 mM	1.25 MV	U87	0.4 to 0.71	0.03 to 0
Miladi et al. [31]	AGuIX, 0.6 mM AGuIX	250 kVp	SQ20B	0.04 to 0.5	0.05 to 0.03
			FaDu	0.01 to 0.2	0.08 to 0.07
			Cal33	−0.05 to 0.07	0.08 to 0.11
	AGuIX, 0.4 mM AGuIX		SQ20B	0.04 to 0.15	0.05 to 0.05
Kotb et al. [62]	AGuIX, 0.6 mg/L AGuIX	220 kVp	B16F10	0.056 to 0.275	0.025 to 0.022
Stewart et al. [63]	Bi_2_O_3_ NP, 50 μg/mL	125 kVp	9 L gliosarcoma cell	0.075 to 0.355	0.017 to 0
		10 MV		0.150 to 0.256	0.013 to 0.009
Wozny et al. [47]	AGuIX, 0.8 mg/mL AGuIX	250 kVp	SQ20B	0.07 to 0.19	0.03 to 0.04
Simonet et al. [54]	AGuIX, 0.8 mM Gd	250 kVp	SQ20B J.L.	0.1593 to 0.2357	0.0079 to 0.0088

Note: The values of *α* and *β* given in reference [62] were not correct. The values here are estimated from the survival curve in Figure 3a of the reference.

## Data Availability

Data sharing not applicable.

## Appendix A

**Table A1:** Technical details of the MC simulations (the checklist item number is in line with TG 268 [64]).

Checklist Item #	Item Name	Description	References
2, 3	Code, version/release date	Geant4, v.10.07.p02/released on 14 June 2021	Ref. [41] http://geant4.web.cern.ch/ accessed on (10 December 2021)
4, 17	Validation	The general Geant4 framework has been validated extensively.	https://geant-val.cern.ch/ accessed on (10 December 2021)
5	Timing	All simulations were performed on an Intel^®^ Xeon(R) CPU E5-2690 v2, with a 64GB memory. In Step 1, each simulation took about 7500 s for 2 × 10^9^ histories. In Step 2, each took about 1800 s for 2 × 10^4^ histories. In Step 3, each took about 1800 s for 5 × 10^5^ histories. In Step 4, each took about 2700 s for 2 × 10^6^ histories.	
8	Source description	The spectra of the parallel beams of 105 kVp, 220 kVp, 250 kVp were generated using SpekCalc. Elekta 6 MV spectrum presented by Sheikh-Bagheri et al. was used for the 6MV source.	Refs. [65,66]
9	Cross-sections	Steps 1 and 3, Livermore package incorporated in Geant4; Step 2: Geant4-DNA option 2. Step 4: Livermore package incorporated in Geant4 was used for photon and electron transport in the NPs, and Geant4-DNA option 2 was used for electron transport in water.	Refs. [42,43,44,45,67]
10	Transport parameters	Steps 1 and 3, the minimum threshold of secondary particle production was used (250 eV); Step 2: tracking cut was set to 7.4 eV; Step 4: tracking cut was set to 7.4 eV for the transport of electrons in water; the minimum threshold of secondary particle production (250 eV) and lowest electron energy of 7.4 eV were used for the transport of electrons in the NPs.	
11	VRT and/or AEIT	Step 1: Geometrical importance sampling was used for the region near the water cylinder center; Step 2: Neither VRT nor AEIT was used; Step 3: physics-based biasing was used to amplify the Compton scattering and photo-electric interaction cross-sections, secondary electrons and photons were killed upon generation; Step 4: the same physics-based biasing as in Step 3 was used for the transport of photons in the NPs.	Ref. [68]
12	Scored quantities	Step 1: number and phase-space data of photons entering the NP-representing sphere, electrons spectrum in the sphere and dose near the sphere; Step 2: the energy deposition of electrons in water; Step 3: the number of ionizations in an NP and the number of photons entering the NP; Step 4: the number of ionizations and the energy deposition of secondary electrons in the nucleus.	Ref. [69]
13, 18	# of histories/statistical uncertainty	To achieve <2% relative uncertainty for the quantities to calculate, 2 × 10^9^, 2 × 10^4^, 5 × 10^6^, and 2 × 10^6^ histories were used for the simulations in Steps 1, 2, 3, and 4, respectively.	
14	Statistical methods	The history-by-history method was used.	Ref. [70]
15, 16	Postprocessing	See Section 2.6 for details.	
